# “O.R. GOES GREEN”: a first step toward reducing our carbon footprint in the operating room and hospital

**DOI:** 10.1007/s13304-024-01793-8

**Published:** 2024-03-25

**Authors:** Nicola Leone, Gitana Scozzari, Francesco Olandese, Tim Horeman, Roberto Passera, Alberto Arezzo, Mario Morino

**Affiliations:** 1 Department of Surgical Sciences, University of Turin, C.So Dogliotti 14, 10126 Turin, Italy; 2 Department of Health Care Management, Città della Salute e Delle Scienze Molinette, Turin, Italy; 3 Department of Biomechanical Engineering, Technical University of Delft, Delft, The Netherlands; 4 Department of Medical Sciences, University of Torino, Turin, Italy

**Keywords:** Carbon footprint, Environment pollution, Hospital waste, Biohazardous waste, Separate collection waste, Operating room

## Abstract

Hospitals in Europe produce approximately 6 million tons of medical waste annually, about one-third of this originating in operating rooms. Most of it is solid waste, which can be recycled if bodily fluids do not contaminate it. Only 2–3% of hospital waste must be disposed of as infectious waste, and this is much lower than the 50–70% of garbage in the biohazard waste stream. In June 2021, at the main operating room of the Department of General Surgery of the University of Turin, we began a separate collection program for materials consisting of plastic, paper, TNT (material not contaminated by bodily fluids), and biohazardous waste. We calculated the number of boxes and the weight of special waste disposed produced every month in one operating room for 18 months. The monthly number of Sanibox and the monthly weight of biohazardous waste decreased during the observation period. The reduction trend was not constant but showed variations during the 18 months. Direct proportionality between number of low-complexity procedures and production of biohazardous waste was found (*p* = 0.050). We observed an optimization in the collection and filling of plastic, paper and TNT boxes separated and sent for recycling. One of the barriers to recycling hospital waste, and surgical waste in particular, is the failure to separate infectious waste from clean waste. A careful separate collection of waste in the operating room is the first step in reducing environmental pollution and management costs for the disposal of hospital waste.

## Introduction

Hospitals in Europe produce approximately 6 million tons of medical waste annually [1] about one-third of this originating in operating rooms. In the United States, hospitals rank second in waste generation, with more than 4 billion tons of waste produced in a year [2]. Infect, the National Health System (NHS) is responsible for 8–10% of total greenhouse gas emissions [3, 4]. All of this affects climate change, which is a major threat to public health in the twenty-first century [5]. Has been estimated that the impact in annual CO_2_ production in an operating theatre in a university hospital in the UK is equivalent to the production generated by the energy consumption of more than 2000 homes [6]. Surgery is an equipment-intensive and waste-intensive specialty since all equipment must be sterile to various standards. For this reason, the trend has been toward an increase in disposable equipment—though other choices often exist, including reusable and reprocessing equipment. Many types of equipment may be purchased in a disposable or reusable form. Traditionally, the choice focused on cost, patient safety, efficacy and ease of use—however, it has not included environmental considerations. Disposable equipment has become increasingly popular because they easily eliminate the risk of cross-contamination between patients. Hospitals are the largest emitters of carbon dioxide, so greener health care will have a great positive impact on the environment [7].

More recent estimates suggest that operating rooms are responsible for 60–70% [8, 9] of hospital waste. The packaging material used to protect and maintain the sterility of supplies and equipment accounts for a large part of the waste. Also, the increased use of disposable supplies and equipment contributes to the problem. Infectious material, sharps and certain medications, which are hazardous to the environment, must be discarded into special containers and fall under the category of Regulated Medical Waste (RMW).

Most of the material generated in the operating room (OR) is solid waste, which can be recycled if bodily fluids do not contaminate it. One of the barriers to recycling is the failure to separate infectious waste from clean waste. Concern regarding infectious contamination has been one of the most significant barriers to fully capturing recyclable material from the OR. However, with only the recycling of non-contaminated material, the share of biohazardous waste could be reduced. Many pieces of surgical equipment and anaesthesia supplies are made from recyclable materials such as plastic, paper, glass and metal. Most of the general operating room waste can be recycled by educating operating room staff on how to properly separate and dispose of this material.

Interest in the reuse or recycling of medical waste is growing globally [10, 11]. In fact, increasingly medical centres are attempting to establish recycling programs for material product in the operating rooms and medical procedure units. This is not only to have environmental benefits, but also because reducing hospital waste disposal costs saves money, generating potential financial savings for hospitals [12]. The dissemination within surgical eques of knowledge about the carbon footprint resulting from their work and how it can be reduced is necessary [13].

Therefore, by modifying the collection and disposal of room material, generating containers of recoverable materials, we expect to obtain considerable economic savings for the hospital but above all a reduction in the environmental impact. In fact, biohazardous waste is mainly disposed of in incinerators that produce increased CO_2_ emission. According to the Environmental Protection Agency (EPA), incinerated medical waste is a major source of dioxins and the main polluter of mercury [14, 15]. Reducing this type of waste would notably reduce the carbon footprint.

In Italy, the management of hospital waste is regulated by Presidential Decree DPR 254/2003. This represents the implementing regulation of Legislative Decree 22/1997. Hazardous medical waste with an infectious risk must be disposed of by thermal destruction in authorized facilities, as stipulated in Article 10. In April 2006, Legislative Decree D.LGS 152/2006 came into force and states that waste management is revaluated from the perspective of European directives. According to this decree, it is the responsibility of public administrations to promote the life cycle analysis of products based on uniform methodologies.

However, the climate emergency requires the implementation of effective measures to reduce the environmental impact of the health care system, which has become one of the main goals of the 2021 COP26 (XXVI United Nations Climate Change Conference, Glasgow 2021) [16].

This manuscript presents the results of a pilot study conducted in our Department of General Surgery at the University of Turin (Italy) aimed at implementing careful waste collection and separation in the operating room, with the objective of evaluating the extent of biohazard waste reduction achieved by a mere behavioural modification.

## Materials and methods

This pilot project was executed in collaboration with the Technical University Delft and OR waste recycling consortium “GreenCycl” in the Netherlands. This collaboration provided practical examples for the creation of our “Green Team” and provided guidelines about recognizing and selecting valuable waste streams and to disseminate knowledge to the OR staff and sterilization department [17].

We formed our “Green Team”, a group of professional figures (two surgeon, one anesthesiologist, one head nurse, two hospital managers) who possessed the knowledge about sustainability. The Green Team's program was based on five pillars, or "5Rs": Reduce, Reuse, Recycle, Rethink and Research [18]. The "Green Team”, through meetings and dissemination of materials explained to the OR staff the various procedures to be performed and implemented for proper waste sorting in the operating room. The “Green Team” also was constantly updating and held meetings to keep all the OR staff updated.

In June 2021, at a single operating room of the Department of General Surgery, we started a separate collection program for materials consisting of plastic, paper, TNT (non-woven fabric), material not contaminated by bodily fluids, and biohazardous material (disposed of in containers called "Sanibox"). All bins had the same capacity (60 L) and have been arranged for the differentiated collection of materials within the entire operating room. Signs have been placed, on each bin, with indications on which material to dispose of in each of them. The separated waste was disposed of not as hospital waste but following the recycling chain.

The number of containers and their weight were counted day by day as well as the surgical activity and the type of procedures performed.

In the 18 months of observation, from June 2021 to November 2022, data were collected regarding the numbers of containers filled with the different waste materials produced in our main operating room, carefully separated: plastic, paper, TNT, non-recyclable material with body fluid contamination disposed of in special containers (Sanibox) as required by law (RMW). The weight of the Sanibox was provided by the company responsible for the disposal of biohazardous medical waste.

An analysis was also conducted on the number and type of surgeries performed. Throughout the observation period, the surgeries procedures performed were 1369. The procedures were divided into high complexity 520 (hemicolectomy, hepatectomy, pancreatectomy, rectum anterior resection, sleeve gastrectomy, gastric bypass, etc.), medium complexity 359 (cholecystectomy, incisional hernia repair, small bowel resection, gastric fundoplication, Heller-Dor procedure, etc.) and low complexity 490 (hernia repair, appendectomy, anal fistulectomy, hemorrhoidectomy, etc.) surgeries. Laparoscopy and laparotomy procedures were included. Robotic procedures were not included. We have divided the surgeries solely according to complexity, because for each degree of complexity the material used in the OR is almost standardized. The intraoperative protocols and materials used were not changed during this period. The health personnel involved has always been the same.

As for descriptive statistics, the categorical variables were reported as absolute/relative frequencies, while the continuous ones as median/IQR (Inter Quartile Range). As for inferential analyses, the bins collection over time have been considered as independent data and, after having recoded as tertiles both the months of collections and the different complexity procedures the Kruskal–Wallis test (a non-parametric analysis of variance) was applied.

All p-values were obtained using the two-sided exact method, at the conventional 5% significance level. Data were analyzed as of November 2023 by R 4.3.2 (R Foundation for Statistical Computing, Vienna-A, http://www.R-project.org).

Due to its explorative nature, no formal sample size determination has been planned for this pilot study. Due to the observational nature of this research, and in accordance with Italian law (Agenzia Italiana del Farmaco-AIFA, Guidelines for observational studies, 20 March 2008), no formal approval from the local Institutional Review Board/Independent Ethics Committee was needed.

## Results

**Fig. 1:**
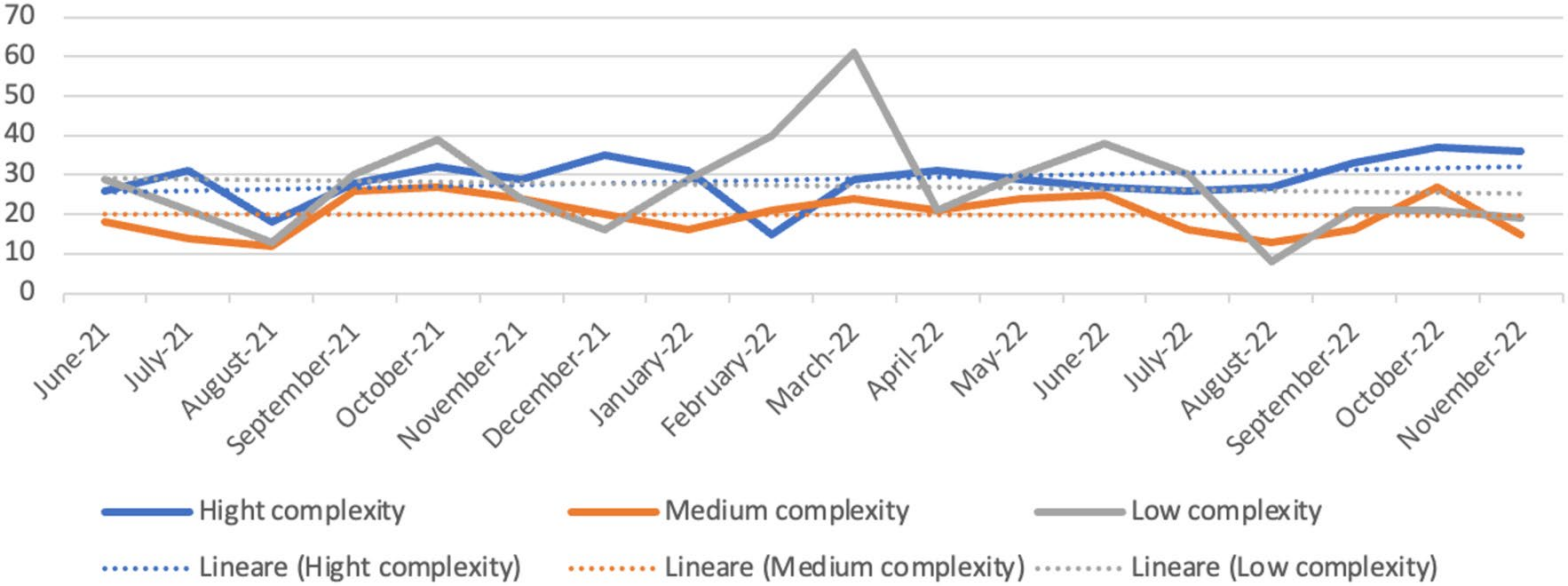
Surgical procedures with trendies indicating no relevant trends that can influence statistics

The project started in June 2021. The number and percentage of the surgical procedures (Table 1) has been almost constant during these 18 months except for low complexity surgical interventions, which saw their maximum peaks in October 2021, February, March and June 2022 (Fig. 1).

**Table 1:** Surgical procedures separated as low, middle and high complexity predicted over time (number and percentage)

Date	N high complexity (%)	N medium complexity (%)	N low complexity (%)
June-21	26 (35.6)	18 (24.7)	29 (39.7)
July-21	31(47.0)	14 (21.2)	21 (31.8)
August-21	18 (41.9)	12 (27.9)	13 (30.2)
September-21	28 (33.3)	26 (31.0)	30 (35.7)
October-21	32 (32.7)	27 (27.6)	39 (39.8)
November-21	29 (37.7)	24 (31.2)	24 (31.2)
December-21	35 (49.3)	20 (28.2)	16 (22.5)
January-22	31 (40.8)	16 (21.1)	29 (38.2)
February-22	15 (19.7)	21 (27.6)	40 (52.6)
March-22	29 (25.4)	24 (21.1)	61 (53.5)
April-22	31 (42.5)	21 (28.8)	21 (28.8)
May-22	29 (34.9)	24 (28.9)	30 (36.1)
June-22	27 (30.0)	25 (27.8)	38 (42.2)
July-22	26 (36.6)	16 (22.5)	29 (40.8)
August-22	27 (56.3)	13 (27.1)	8 (16.7)
September-22	33 (47.1)	16 (22.9)	21 (30.0)
October-22	37 (43.5)	27 (31.8)	21 (24.7)
November-22	36 (51.4)	15 (21.4)	19 (27.1)

**Fig. 2:**
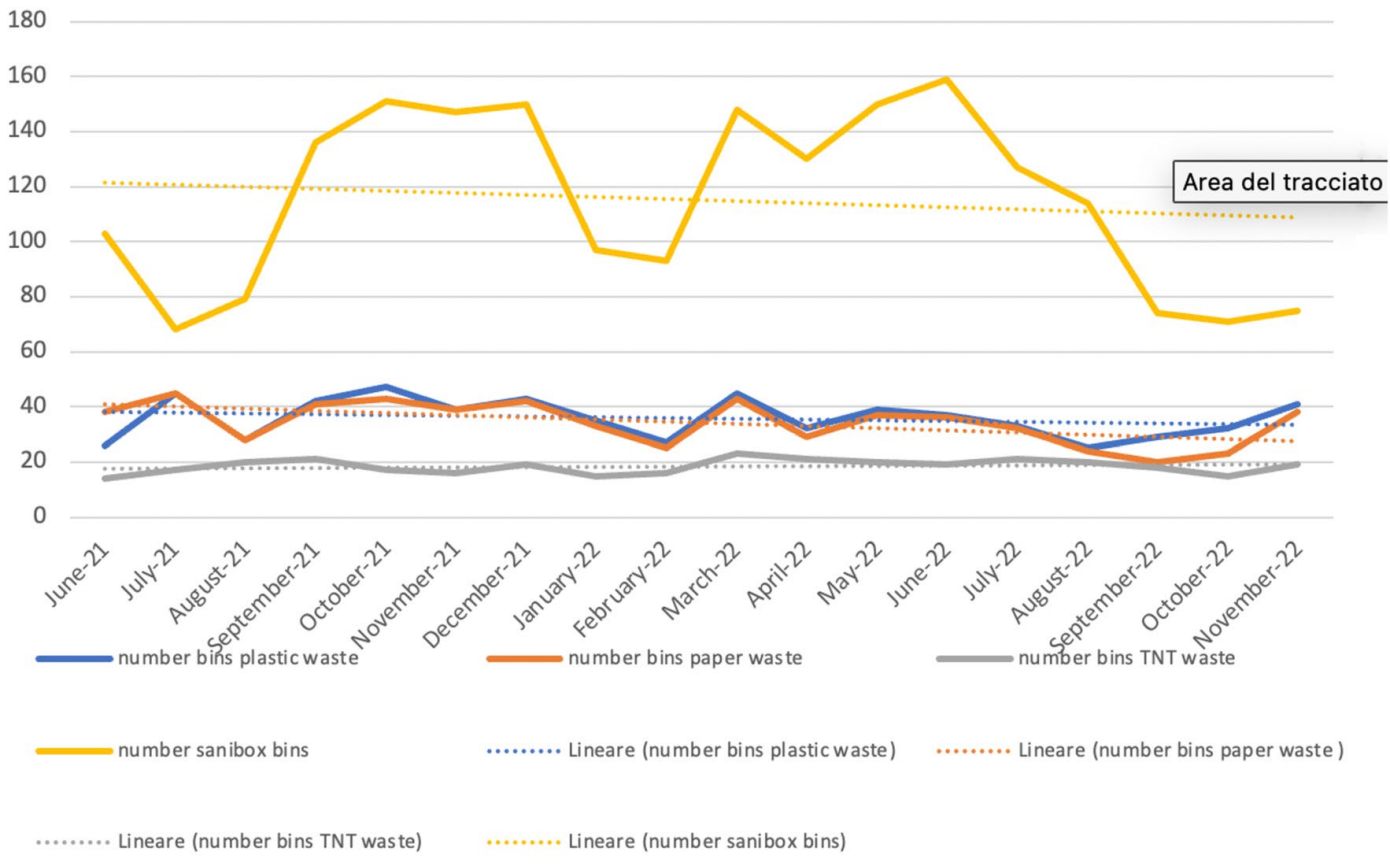
Number bins of collected waste with trendlines indicating no relevant trends that can influence statistics

**Fig. 3:**
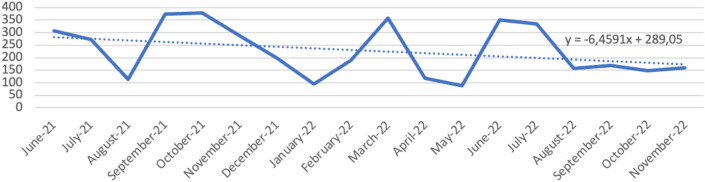
Sanibox weight (Kg) with trendline showing a small decline in number of waste bins during the pilot

The monthly number of Sanibox and the monthly weight of biohazardous waste decreased during the observation period (Table 2). The reduction trend was not constant but showed variations during the 18 months (Figs. 2, 3). The median number of boxes of special waste disposed passed from 120 in the first tertile (0–6 months) to 95 in the third tertile (13–18 months). Assessing the median in the three tertiles shows a reduction in the third tertile, but not statistically significant (*p* = 0.481). The median monthly weight of the same waste decreased from 297,06 kg in the first tertile (0–6 months) to 164.30 kg in the third tertile (13–18 months). This decrease is also not constant and not statistically significant (*p* = 0.238) (Table 3). This was the waste disposed of as regulated medical waste. This reduction is more evident in the last months of the period under examination (August–November 2022) as it became necessary to correctly and meticulously disseminate the guidelines to be followed. Similarly, there was a reduction in the median number of paper bins (*p* = 0.049). The median number of plastic bins had an interesting reduction trend but without statistical association (*p* = 0.456). The median number of TNT bins remained constant over time.

**Table 2:** Separated collected waste: number of plastic bins, number of paper bins, number of TNT bins, number of Sanibox bins and Sanibox weight

Date	Number bins plastic waste	Number bins paper waste	Number bins TNT waste	Number Sanibox bins	Sanibox weight (Kg)
June-21	26	38	14	103	306.15
July-21	45	45	17	68	271.74
August-21	28	28	20	79	113.19
September-21	42	41	21	136	373.5
October-21	47	43	17	151	377.95
November-21	39	39	16	147	287.97
December-21	43	42	19	150	199.92
January-22	35	33	15	97	96
February-22	27	25	16	93	189.16
March-22	45	43	23	148	357.26
April-22	32	29	21	130	117.36
May-22	39	37	20	150	87.31
June-22	37	36	19	159	350.69
July-22	33	32	21	127	335.6
August-22	25	24	20	114	157.43
September-22	29	20	18	74	167.91
October-22	32	23	15	71	148.56
November-22	41	38	19	75	160.69

**Table 3:** Number of bins, weight sanibox and surgical procedures during the 18 months recoded as tertiles

	0–6 months					7–12 months					13–18 months				
	Minimum	Percentile 25	Median	Percentile 75	Maximum	Minimum	Percentile 25	Median	Percentile 75	Maximum	Minimum	Percentile 25	Median	Percentile 75	Maximum
Plastic	26	28	41	45	47	27	32	37	43	45	25	29	33	37	41
Paper	28	38	40	43	45	25	29	35	42	43	20	23	28	36	38
Tnt	14	16	17	20	21	15	16	20	21	23	15	18	19	20	21
Sanibox	68	79	120	147	151	93	97	139	150	150	71	74	95	127	159
Weight	113.19	271.74	297.06	373.5	377.95	87.31	96	153.26	199.92	357.26	148.56	157.43	164.3	335.6	350.69
High_compl	18	26	29	31	32	15	29	30	31	35	26	27	30	36	37
Medium_compl	12	14	21	26	27	16	20	21	24	24	13	15	16	25	27
Low_compl	13	21	27	30	39	16	21	30	40	61	8	19	21	30	38

Peaks of production of number and weight of Sanibox bins were observed in those months in which more low complexity procedures were completed. This would suggest a direct proportionality between number of low-complexity procedures and production of biohazardous waste.

Very interesting is what emerged from the stratified analysis of the complexity of surgical procedures (Tables 4, 5, 6).

**Table 4:** Number and wight of sanibox bins in high complexity surgical procedures recoded as tertiles

	Tertile 1					Tertile 2					Tertile 3				
	Minimum	Percentile 25	Median	Percentile 75	Maximum	Minimum	Percentile 25	Median	Percentile 75	Maximum	Minimum	Percentile 25	Median	Percentile 75	Maximum
Plastic	25	26	28	33	37	35	39	41	45	45	29	32	37	43	47
Paper	24	25	30	36	38	33	37	40	43	45	20	23	34	42	43
Tnt	14	16	20	20	21	15	16	19	21	23	15	17	19	19	21
Sanibox	79	93	109	127	159	68	97	142	148	150	71	74	103	150	151
Weight	113.19	157.43	247.65	335.6	350.69	87.31	96	279.86	357.26	373.5	117.36	148.56	164.3	199.92	366.95

**Table 5:** Number and wight of sanibox bins in medium complexity surgical procedures recoded as tertiles

	Tertile 1					Tertile 2					Tertile 3				
	Minimum	Percentile 25	Median	Percentile 75	Maximum	Minimum	Percentile 25	Median	Percentile 75	Maximum	Minimum	Percentile 25	Median	Percentile 75	Maximum
Plastic	25	28	33	41	45	26	27	39	43	45	32	35	40	45	47
Paper	20	24	32	38	45	25	29	38	42	43	23	30	39	42	43
Tnt	15	17	19	20	21	14	16	19	21	23	15	16	18	20	21
Sanibox	68	74	79	114	127	93	103	147	150	150	71	104	144	155	159
Weight	96	113.19	160.69	271.74	335.6	87.31	117.36	199.92	306.15	357.26	148.56	249.63	362.1	375.73	377.95

**Table 6:** Number and wight of sanibox bins in low complexity surgical procedures recoded as tertiles

	Tertile 1					Tertile 2					Tertile 3				
	Minimum	Percentile 25	Median	Percentile 75	Maximum	Minimum	Percentile 25	Median	Percentile 75	Maximum	Minimum	Percentile 25	Median	Percentile 75	Maximum
Plastic	25	29	32	42	45	26	33	37	39	42	27	32	41	46	47
Paper	20	24	29	40	45	32	33	38	39	41	25	31	40	43	43
Tnt	15	18	19	20	21	14	15	18	21	21	16	17	18	21	23
Sanibox	68	73	77	122	150	97	103	132	147	150	93	121	150	155	159
Weight	113.19	132.96	159.06	183.92	271.74	87.31	96	297.06	336.6	373.5	189.16	269.93	353.98	367.61	377.95

**Fig. 4:**
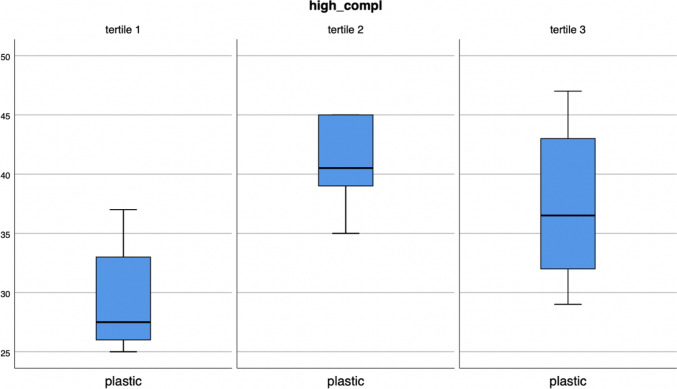
Plastic bins in high complexity surgical procedures recoded as tertiles (*p* = 0.012)

Considering high complexity surgeries, plastic waste decreases as the number of these surgeries decreases (*p* = 0.012) (Fig. 4). For the other types of waste, no statistical association has been found.

**Fig. 5:**
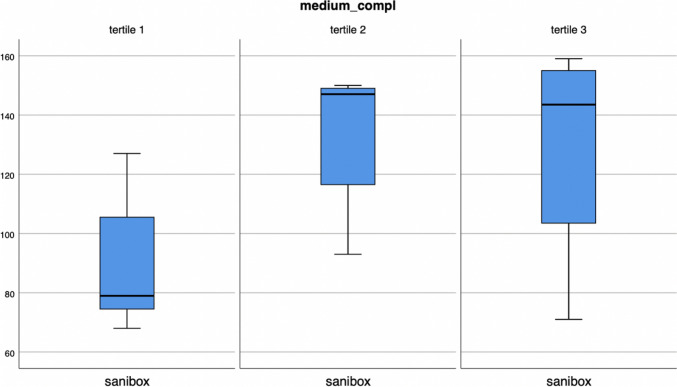
Sanibox in medium complexity surgical procedures recoded as tertiles (*p* = 0.030)

For medium-complexity surgeries, as their number decreases, there is a statistically significant reduction in the number of Saniboxes (*p* = 0.034) (Fig. 5) and a reduction in the weight of biohazardous waste, even not statistically supported (*p* = 0.156).

**Fig. 6:**
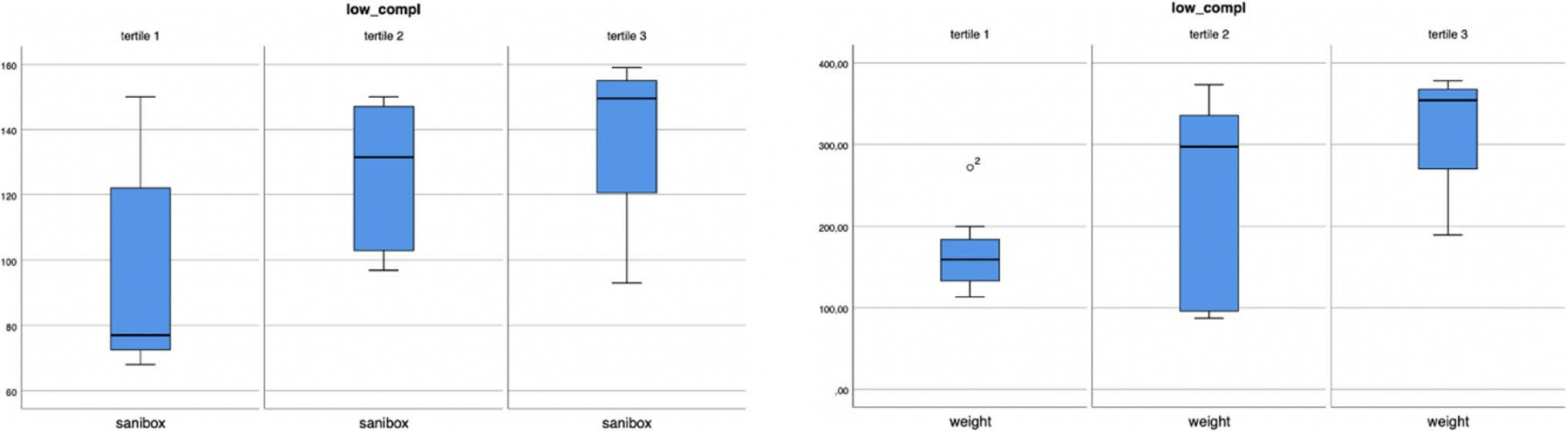
Number and weight of sanibox in low complexity surgical procedures recoded as tertiles (*p* = 0.046; *p* = 0.050)

For last category, a direct association was observed between the number of low-complexity surgeries and other the number of Saniboxes (*p* = 0.046), or the weight of biohazardous waste (*p* = 0.050) (Fig. 6).

## Discussion

Surgical waste is a worldwide growing phenomenon, increasing CO_2_ emissions. The growing carbon footprint results in climate changes such as global warming that impacts public health primarily through the redistribution of infectious diseases. By 2030, the World Health Organization (WHO) has predicted 250,000 more deaths per year because of climate change [5].

Each surgical operation generates 3.5–8.4 kg [19, 20] of plastic waste with 20% high-quality polypropylene (PP). The increasing number of surgical interventions adds to millions of kilograms of surgical waste yearly [21, 22]. Moreover, surgical waste causes a significant financial burden for hospitals (depending on waste processing contracts). In our hospital, "A.O.U. Città della Salute e della Scienza di Torino (Molinette)", the price per kg for the disposal service of biohazardous waste is equal to 1.24 euro/kg + VAT. Due to lacking knowledge of circular waste management in healthcare, the waste accumulation problem is rarely addressed [10, 12, 23]. Hospitals manage their medical waste in different ways and any coordination program is fading, where there would be a need to adopt shared and "virtuous" measures. Consequently, technical innovation for optimal and shared surgical waste recycling and socio-economic analysis monitoring is necessary to accelerate the circular transition in the healthcare domain. Unfortunately, at present, there are still few projects and actions to achieve this goal.

Furthermore, medical waste poses a potential threat to employees, patients, and surrounding communities [24]. It is, therefore, important to reduce the volume of medical waste to reduce potentially dangerous effects [19]. To do this, it is necessary to develop a “circular economy” program which reduces biohazardous waste by promoting the recycling of non-contaminated materials. Doing so would not only reduce environmental pollution but also reduce the cost of waste management by hospitals [25, 26]. Within our pilot study, we reduced the processing and incineration costs related to biohazard fees with 3483.41 Euro + VAT. At the same time, we reduced the CO_2_ emission (CO_2_e) significantly. 1 kg of biohazardous waste generates approximately 2.8 kg of CO_2_e when being transported and incinerated [25]. Since in total 2809.2 kg was saved from incineration during the pilot, 1265.04 kg of CO_2_e was saved. Each kilo of plastic, paper and TNT that is recycled saves approximately 0.5 kg, 0.15 kg, and 0.58 kg of CO_2_e, respectively. If we now look at the total amount of plastic, paper and TNT saved from incineration with 580.5 kg, 739.2 kg, and 1489.5 kg, respectively, we saved 290.25 kg, 110.88 kg, and 863.91 kg of CO_2_e in total related to using recycled plastics and paper for new products by hospital mining (Table 7). This emerged from a single operating room. More significant results would be obtained if we applied this protocol to all operating rooms of our hospital.

**Table 7:** Saved CO_2_ emission (CO_2_e) calculated on total number of the TNT, plastic and paper bins

Type waste	Number bins	Volume (M3)	Volume efficiency factor bin (1)	Density (kg/m3)	Kg	Recycling vs raw footprint reduction CO_2_ factor	Saved CO_2_e
TNT	331	19.86	9.93	150	1489.5	0.58	863.91
Plastic	645	38.7	19.35	30	580.5	0.5	290.25
Paper	616	36.96	18.48	40	739.2	0.15	110.88
Total	1592	95.52			2809.2		1265.04

The support of the hospital health management is essential, which by sharing and supporting the project can provide legislative indications, economic feedback and monitor the complex “assembly chain” (from the collection and sorting of waste in the operating room to its disposal). By virtue of all this, we have initiated a national pilot project on this issue.

As a first step, we undertook a web meeting with Delft University and GreenCycle, pioneers in this field, from which we exported all their experience and organization. Subsequently, we established a “Green Team”, which sensitized the operating room staff on the subject and suggested and followed the implementation of simple measures in waste management without additional costs on their management. We found of primary importance to have regular meetings between "Green Team" members. During these meetings there were updates, exchanges of knowledge and opinions, feedback on the work done. Aiming at the reduction of the production of biohazardous waste favored by a careful and scrupulous differentiated collection in the operating theatre. The “Green Team” is essential for carrying out this activity, as coordination and as a group of professionals who are always up to date and ready to intervene in case of problems [7]. This led to a constant and progressive reduction in the production of biohazardous waste, mainly in the last months of the study, by which time the system had run in, and all the staff knew what to do and how to do it. Separate waste collection in the operating room and minimum effort on the part of all staff have promoted a reduction in environmental pollution and economic savings on the part of the hospital. All of this was done without jeopardizing the safety of patients and staff, without changing treatment strategies and the types of operations performed. The behavioral changes were welcomed by all health care staff with enthusiasm and interest, as the changes were minimal for a major achievement. In addition, the outreach work of the “Green Team” was of paramount importance to achieve this.

Another point to discuss and on which much awareness is needed is the optimization of the filling of the Sanibox. At the beginning of the study, as in previous years, the Sanibox were closed and disposed of even if not full. At the end of the period under review, Sanibox decreased significantly compared to the previous number, although not reaching statistical significance (*p* = 0.482).

In addition, we looked at the types and numbers of surgeries performed, which did not change during the 18 months of observation. A direct proportionality has emerged between the increase in minor surgery and number of the sanibox and biohazardous waste (*p* = 0.046; *p* = 0.050). In fact, minor surgeries are shorter and consequently, the number of those performed in a day is higher. This leads to increased waste, mainly contaminated waste, favouring increased biohazardous waste. Most of the contaminated waste consists of surgical drapes (drapes placed on the patient), gauze and everything needed to monitor the patient's cardio-circulatory activity during surgery. Major surgical interventions, having a longer duration and not requiring continuous changes of the patient's drapes.

Several studies in the literature emphasize how crucial it is to reduce the environmental pollution generated in the operating room [2] and how effective it can be to address the problem "from within." [25]. There are still no studies that show clear and concrete data as reported here.

The carbon footprint generated by the disposal of hospital waste is a very current problem. Throughout the world, efforts are being made to change the attitude of operating room staff and encourage the recycling of materials not contaminated by biological fluids thus reducing CO_2_e [26]. Providing clear numerical data such as those presented could further raise awareness among this topic all healthcare professionals.

This study has several intrinsic limitations. It is a retrospective observational analysis based only on data from a single operating room over a short period of time. In addition, it was partly conditioned by the contextual Covid pandemic, which in some months forced a considerable reduction in surgical activity. Nevertheless, it represents aim interesting and encouraging starting point because it demonstrates that with the differentiation of non-contaminated material it is possible to obtain a reduction of biohazardous waste.

## Conclusion

Environmental pollution is a global problem that must be tackled seriously and drastically. Hospital waste, especially biohazard waste, largely contributes to carbon footprint by changing the Earth's climate structure and contributing to the cost of its disposal. Within a short pilot demonstrated that minor surgery procedure directly contributes significantly to the biohazardous waste mass and volume. In our experience, it has emerged that behaviour change in the operating room can be established by introducing a recycling program. This drastically reduced the CO_2_e footprint related to incineration of biohazardous waste, while promoting economic savings for the hospitals.

## Data Availability

The data in the study are available for consultation.
